# Comparative Investigation of Copper Tolerance and Identification of Putative Tolerance Related Genes in Tardigrades

**DOI:** 10.3389/fphys.2017.00095

**Published:** 2017-02-28

**Authors:** Thomas L. Hygum, Dannie Fobian, Maria Kamilari, Aslak Jørgensen, Morten Schiøtt, Martin Grosell, Nadja Møbjerg

**Affiliations:** ^1^Section for Cell Biology and Physiology, Department of Biology, University of Copenhagen Copenhagen, Denmark; ^2^Centre for Social Evolution, Department of Biology, University of Copenhagen Copenhagen, Denmark; ^3^Marine Biology and Fisheries, Rosenstiel School of Marine and Atmospheric Science, University of Miami Miami, FL, USA

**Keywords:** chemobiosis, copper tolerance, cyst, toxicants, osmoregulation, stress tolerance, tardigrades, transcriptome

## Abstract

Tardigrades are microscopic aquatic animals renowned for their tolerance toward extreme environmental conditions. The current study is the first to investigate their tolerance toward heavy metals and we present a novel tardigrade toxicant tolerance assay based on activity assessments as a measure of survival. Specifically, we compare tolerance toward copper in four species representing different evolutionary lineages, habitats and adaptation strategies, i.e., a marine heterotardigrade, *Echiniscoides sigismundi*, a limno-terrestrial heterotardigrade, *Echiniscus testudo*, a limno-terrestrial eutardigrade, *Ramazzottius oberhaeuseri*, and a marine eutardigrade, *Halobiotus crispae*. The latter was sampled at a time of year, when the population is predominantly represented by aberrant P1 cysts, while the other species were in normal active states prior to exposure. Based on volume measurements and a general relation between body mass and copper tolerance, expected tardigrade EC50 values were estimated at 0.5–2 μg l^−1^. Following 24 h of exposure, tolerance was high with no apparent link to lineage or habitat. EC50s (95% CI), 24 h after exposure, were estimated at 178 (168–186) and 310 (295–328) μg l^−1^, respectively, for *E. sigismundi* and *R. oberhaeuseri*, whereas *E. testudo* and *H. crispae* were less affected. Highest tolerance was observed in *H. crispae* with a *mean* ± *s.e.m*. activity of 77 ± 2% (*n* = 3) 24 h after removal from ~3 mg l^−1^ copper, suggesting that tardigrade cysts have increased tolerance toward toxicants. In order to identify putative tolerance related genes, an *E. sigismundi* transcriptome was searched for key enzymes involved in osmoregulation, antioxidant defense and copper metabolism. We found high expression of Na/K ATPase and carbonic anhydrase, known targets for copper. Our transcriptome, furthermore, revealed high expression of antioxidant enzymes, copper transporters, ATOX1, and a Cu-ATPase. In summary, our results indicate that tardigrades express well-known key osmoregulatory enzymes, supporting the hypothesis that copper inhibits sodium turnover as demonstrated for other aquatic organisms. Tardigrades, nevertheless, have high tolerance toward the toxicant, which is likely linked to high expression of antioxidant enzymes and an ability to enter dormant states. Tardigrades, furthermore, seem to have a well-developed battery of cuproproteins involved in copper homeostasis, providing basis for active copper sequestering and excretion.

## Introduction

Tardigrades are microscopic metazoans that require a film of water to be in an active and reproducing state. These minute aquatic animals have adapted to terrestrial environments by evolving dormant states, cryptobiosis, and cysts, and they are famous for their ability to survive a range of environmental extremes, including complete desiccation, high levels of radiation, extreme hydrostatic, and osmotic pressures, very low sub-zero temperatures as well as space conditions (e.g., Rebecchi et al., 2007; Jönsson et al., 2008; Hengherr et al., 2009; Guidetti et al., 2011; Møbjerg et al., 2011; Persson et al., 2011; Welnicz et al., 2011; Clausen et al., 2014; Zawierucha et al., 2015; Hashimoto et al., 2016; Heidemann et al., 2016; Hygum et al., 2016). Nevertheless, little is currently known about tardigrade physiology and very few studies exist on tardigrade tolerance toward environmental toxicants (Sobczyk et al., 2015).

Here, we hypothesize that tardigrades, due to their unique adaptations, have a high tolerance toward toxicants. In the current study, we focus on copper, a potent toxicant commonly found in aquatic environments as a consequence of anthropogenic activities (Eisler, 1998; Grosell et al., 2002, 2007). The metal exerts its toxic effects by inhibiting osmoregulatory enzymes and by inducing oxidative stress (Grosell et al., 2002; Lushchak, 2011). Copper-tolerance has been linked to sodium turnover rates, which are strongly related to body size (Grosell et al., 2007). Specifically, copper interferes with sodium metabolism by interacting with key enzymes, such as the Na/K ATPase and carbonic anhydrase (e.g., Vitale et al., 1999; Esbaugh et al., 2015). Thus, aquatic invertebrates with high integument permeability or small size seem to be particularly sensitive to the deleterious effect of the metal ion. In addition, copper, due to its variable valencies, promotes the formation of free radicals leading to oxidative stress (e.g., Balamurugan and Schaffner, 2006).

Given the small size of tardigrades, and thus their high surface area to volume ratio, one would expect them to be relatively sensitive to copper exposure. On the other hand, due to their unique adaptations to environmental extremes, they could have a far greater tolerance than expected from size alone. Tardigrades (phylum Tardigrada) are divided into two main extant groups represented by the classes Heterotardigrada and Eutardigrada. Species living on land need a film of water to be active, and are thus referred to as limno-terrestrial or semi-terrestrial. The species chosen for the current investigation represent these two evolutionary lineages, as well as different habitats and adaptation strategies. Specifically, the study compiles data on copper tolerance in two heterotardigrades, i.e., the limno-terrestrial *Echiniscus testudo* (Doyére, 1840) and the marine-tidal *Echiniscoides sigismundi* (Schultze, 1865), as well as two eutardigrades, i.e., the limno-terrestrial *Ramazzottius oberhaeuseri* (Doyère, 1840), and the marine *Halobiotus crispae* Kristensen, 1982. The latter species, uniquely, has cyclomorphosis involving formation of an aberrant cyst (Kristensen, 1983; Møbjerg et al., 2007; Halberg et al., 2009).

Our data on tardigrade copper tolerance is evaluated and discussed in relation to (i) the first transcriptome data on a heterotardigrade, i.e., *E. sigismundi*, in conjunction with existing genome and transcriptome data from, among others, eutardigrades, as well as (ii) previously published EC50 data from selected invertebrate species, i.e., freshwater mussels of the family Unionidae and the more closely related crustaceans, *Daphnia magna* Strauss, 1820 and *Artemia franciscana* Kellogg, 1906. These invertebrate species show a rather large variation in EC50 values for copper, ranging from 14 to 57 μg l^−1^ in Unionidae (Wang et al., 2007) over 68 μg l^−1^ in *A. franciscana* (Brix et al., 2006; assay testing hatching success rather than mortality) to 424 μg l^−1^ in *D. magna* (De Schamphelaere et al., 2002). Due to the close evolutionary proximity to crustaceans as well as a similarity in size one could expect tardigrade tolerance to fall within the range of the brine shrimp *A. franciscana*.

## Materials and methods

### Tardigrade collection and handling

#### Marine tardigrades

The marine heterotardigrade *E. sigismundi* was collected in May–June 2014 on barnacles in the intertidal zone at Lynæs, Zealand, Denmark (55° 56′ 52.3″ N, 11° 51′ 07.8″ E). The sampling site at Lynæs is a tidal beach area with brackish water and a measured salinity around 18 ppt during the samplings. Barnacle shells, cleaned from soft tissue, were freshwater shocked and filtered through 500 and 62 μm mesh sieves. The material retained by the latter sieve, containing *E. sigismundi*, was stored at 5°C in seawater from the locality for 4–5 weeks with regular water changes. A total of ~780 *E. sigismundi* specimens were used for the copper tolerance assays.

The marine eutardigrade *H. crispae* was collected at Vellerup Vig, Isefjord, Denmark (55°44′7.25″N, 11°51′30.56″E) in August 2014. The water at this sampling site is also brackish with a measured salinity of about 18 ppt. Sediment samples, containing *H. crispae*, were collected at a depth of ~1–1.5 m from a seabed consisting of coarse sand with patches of eelgrass (*Zostora marina*) and numerous stones covered with macro algae and blue mussels (*Mytilus edulis*). Samples were freshwater shocked and filtered through the two sieves mentioned above. The filtrate was subsequently retransferred to locality seawater and stored at 5°C. *H. crispae* is characterized by the presence of cyclomorphosis, i.e., seasonal cyclic changes in morphology and physiology. The specimens used in the current study were white-colored indicating that they were in the so-called pseudosimplex-1 stage (P1), an aberrant cyst stage characterized by the presence of a double cuticle (e.g., Halberg et al., 2013c). Approximately 750 specimens of this species were used in copper tolerance assays.

#### Limno-terrestrial tardigrades

Two limno-terrestrial tardigrades, the heterotardigrade *E. testudo* and the eutardigrade *R. oberhaeuseri*, were collected from mosses and leaf litter from a roof in Nivå, Denmark (55° 56′ 36.53″ N, 12° 30′ 00.90″ E) during June–July 2014. The moss was cleaned and filtered under freshwater through the two aforementioned sieves. The filtrate containing tardigrades and debris from the mosses was stored at 5°C in ultrapure water (Barnstead EASYpure UV/UF, Dubuque, IA, USA). In total, ~530 specimens of each species were used in the copper tolerance assays.

### Volume measurements

Volumes of single tardigrades were estimated using a compound microscope (Leica DM1000, Wetzlar, Germany) equipped with an InfinityX camera (DeltaPix, Smørum, Denmark). Depending on the species, the tardigrades were transferred in either sea- or purified water to a microscope slide and covered with a coverslip. Care was taken to leave an appropriate amount of water around the specimens in order not to squeeze them. The specimens were subsequently photographed and their volume was calculated based on measurements with the software ImageJ 1.49 (Rasband, 1997–2015), i.e., body volume was calculated as a cylinder, with the height corresponding to the length of the specimen, and the diameter equaling the width between second and third leg pairs (Halberg et al., 2013a).

### Preparation of copper solutions

A 1 g Cu l^−1^ solution (as CuCl_2_, Sigma Aldrich, St. Louis, Missouri, USA) was prepared in ultrapure water. Dilution series were subsequently prepared from this stock-solution using either seawater from the respective sampling locality or purified water, depending on the habitat of the tardigrade species. Water samples were collected for total copper concentration measurements. Upon collection, water samples were acidified by 1% trace metal grade HNO_3_ and subjected to analyses by graphite furnace atomic absorption spectroscopy (Varian Spectra AA 220Z coupled with a GTS 110Z, Varian Medical Systems, Palo Alto, CA, USA) following general standard operating procedures and using certified standards as reference. Matrix interference in samples of elevated salinity was overcome by either dilution for samples of higher copper concentrations or by standard addition protocols for samples of lower concentrations.

### Copper tolerance assay

Prior to copper exposure, single tardigrades were collected from the stock material under microscope (Zeiss Stemi, 2000; Carl Zeiss, Oberkochen, Germany) using a Pasteur glass pipette or an ordinary laboratory pipette with plastic tip. The specimens were transferred to watch glasses containing either purified water (limno-terrestrial tardigrades) or sterile filtered seawater (0.2 μm filter) from the respective sampling locality (marine tardigrades) and stored at 5°C for up to a few days.

Tardigrades were subsequently assigned into groups of ~20 specimens and each group was transferred into one well of a four-well cell culture plate and exposed to 2 ml of a given copper solution. The copper concentration in these solutions ranged from ~0 to 4 mg l^−1^. Initially, the tolerance assay was tested on specimens of *E. sigismundi*, immersed in copper solutions at ~5°C for periods of 2, 24, and 48 h, respectively (data not shown). Based on these preliminary experiments, a 24 h copper exposure period was chosen, as this length of time seemed sufficient to allow deleterious effects of the metal to take place. Only highly active animals were used in the assays.

**Figure 1:**
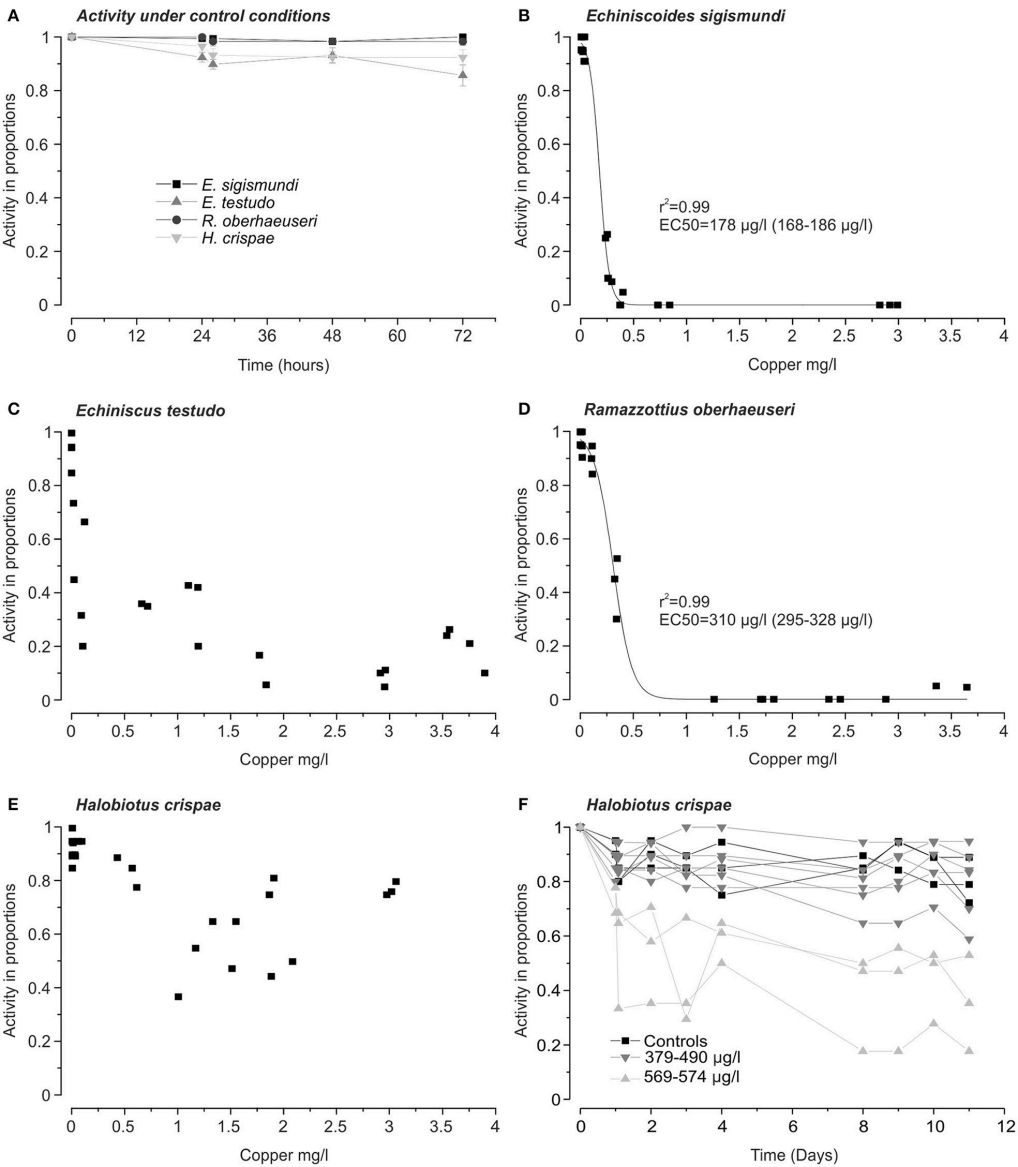
**Activity as a measure of survival in four species of tardigrades following 24 h exposure to copper**. **(A)** Activity in proportions (*mean* ± *s.e.m*.) of each of the four tardigrade species under control conditions (*n* = 9 groups of ~20 *Echiniscoides sigismundi* and *n* = 6 groups of ~20 tardigrades for the other species). **(B–E)** Tardigrade activity 24 h after removal from copper solutions as a function of copper-concentration. Each data-point represents the activity in proportions in a group of ~20 tardigrades exposed to a given measured copper concentration. EC50 values (95% confidence intervals in parentheses) for *E. sigismundi* **(B)** and, *Ramazzottius oberhaeuseri* **(D)** were estimated using a dose-response fit. Fits (not shown) for *Echiniscus testudo* **(C)** and *Halobiotus crispae* **(E)** had low *r*^2^ values due to increased activity rates recorded following exposure to high copper concentrations. **(F)** Activity of *H. crispae* over a period of 10 days following 24 h exposures to copper. Each data-point represents the activity in proportions at a given time for nine groups of ~20 tardigrades exposed to measured copper concentrations of, respectively, 379, 400, 445, 453, 483, 490, 569, 572, and 574 μg l^−1^. For simplicity the 6 groups exposed to the lowest concentrations have been marked by (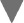), whereas the three groups exposed to the highest concentrations were marked (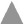). Activity at *t* = 1 day represents the activity of tardigrades immediately following removal from the respective copper-solutions. Three control groups (■) were kept in Vellerup Vig sea water (6 μg Cu l^−1^) for the entire period.

The activity of specimens was assessed by observing the tardigrades in a stereomicroscope at 40–50X magnification (Zeiss Stemi, 2000 or Leica DM1000). Tardigrades often respond to environmental stress by entering cryptobiosis, a dormant state, characterized by metabolic shut-down (Møbjerg et al., 2011). Consequently, in order to avoid low activity counts caused by a cryptobiotic response to the toxicant, activity was assessed following retransfer to habitat water. Thus, following the 24 h copper exposures, specimens were rinsed twice, by transfers into watch glasses containing 2 ml of either locality seawater or purified water, depending on species. After the second wash, specimens were transferred with a new Pasteur-pipette to a third watch glass containing 1.5–2 ml of appropriate water and assessed for activity. Individual specimens were considered active and alive if they exhibited clear movement or responded to tactile stimuli. The specimens were monitored for 48 h after the retransfer with activity checks at 2, 24, and 48 h. For each experimental series, control groups were kept in four-well plates with either locality seawater or purified water (depending on species) and assessed for activity at similar time intervals (Figure 1A). Due to the high tolerance of *H. crispae* an extra set of experiments was performed, in which activity was scored over a period of 10 days following the initial 24 h exposure.

Data on activity is presented as activity in proportions or percentages calculated based on the number of active specimens divided by the total number of specimens in each group. The software OriginPro 9.1 (OriginLab, Northampton, Massachusetts, USA) was used to visualize and analyze correlations between activity and copper-concentration. Specifically, the dose-response regression analysis tool built into Origin was used to estimate EC50 values with 95% threshold confidence intervals, when applicable. Final assemblage of figures was made in CorelDraw X7 (Corel Corporation, Ottawa, Canada).

### Transcriptome analysis of *Echiniscoides sigismundi*

A transcriptome was obtained from a pool of ~550 *E. sigismundi*. Total RNA was extracted using an RNeasy Plus Universal Mini Kit (Qiagen, Hilden, Germany) and quantified using a NanoDrop ND-1000 (Thermo Scientific, Waltham, Massachusetts, USA) and a Bioanalyzer 2100 (Agilent Technologies, Santa Clara, California, USA). The transcriptome was sequenced at BGI (BGI Tech Solutions Co. Shenzhen, Guangdong, China) using Illumina sequencing. Vector-clipping, trimming and quality checking of raw sequence reads yielded ~55 million clean reads. The assembly into contigs using the Trinity software package (Grabherr et al., 2011) and the functional annotation of the Unigenes were performed at BGI. The transcriptome constituted 31,601 unique transcripts (Unigenes) with a total length of ca. 26 million bp and an N50 of 1,524 bp. The protein coding region prediction analysis resulted in a total of 20,937 CDS. Specifically, the number of CDS that mapped to public protein databases was 13,578 and the number of predicted CDS was 7359. The function annotation analysis resulted in 14,159 Unigenes that were annotated with the NR, NT, Swiss-Prot, KEGG, COG, and GO databases. Abundance of each Unigene was reported in FPKM (Fragments per kb of transcript per million mapped fragments; Mortazavi et al., 2008; Trapnell et al., 2010). The mean and median FPKM-values of the 31,601 Unigenes were, respectively, 27.3587 and 4.6156 (FPKM range: 0–4437.8229).

The *E. sigismundi* transcriptome was screened for putative copper sensitive enzymes, i.e., Na/K ATPase and carbonic anhydrase, as well as for transcripts encoding enzymes involved in antioxidant defense and proteins involved in copper transport. Transcripts annotated as such were selected for further analyses using appropriate BLAST searches on both nucleotide and amino acid sequences (cutoff *e* < 0.00001) in NCBI, the *Hypsibius dujardini* (Hd) genome database (Koutsovoulos et al., 2016), the Pfam database (Finn et al., 2016) and Swiss-prot database. The best hit results for each transcript, combining lowest *e*-value and highest identity, were imported into CLC main workbench 6 (CLCbio, Århus, Denmark) and used as query in reciprocal blast searches against the entire *E. sigismundi* transcriptome. Furthermore, reciprocal BLAST searches (BLASTX and TBLASTX using default parameters and a cutoff *e* < 0.00001) were performed between the *E. sigismundi* transcriptome and a number of datasets with relevant genes downloaded from the GenBank EST database (CLC main workbench). The datasets were retrieved and processed after implementing the filter “animals” on September 19th 2016, using the terms “Na/K ATPase,” “carbonic anhydrase,” “glutathione reductase,” “glutathione S-transferase,” “glutathione peroxidase,” “superoxide dismutase,” “metallothionein,” “catalase,” “ATOX1,” “ATP7+ATP7a+ATP7b,” and “copper transporter.” Finally, in order to detect conserved protein regions and document differences between the predicted tardigrade sequences and those of other invertebrates, we downloaded sequences from Flybase (Attrill et al., 2016) and used CLUSTALW (Thompson et al., 1994) to align protein sequences of each relevant gene from *E. sigismundi* with *H. dujardini, Drosophila melanogaster* and the NCBI best hit. The *E. sigismundi* transcripts listed in **Table 3** were selected based on (i) the robustness of these alignments, (ii) transcript length (when applicable the longest transcripts were selected), (iii) number of raw fragments (when applicable transcripts with the most fragments were selected), and (iv) lowest *e*-values obtained during the BLAST searches.

## Results

### Copper tolerance across tardigrade lineages and habitats

Four tardigrade species, *E. sigismundi, E. testudo, H. crispae*, and *R. oberhaeuseri*, representing different evolutionary lineages, habitats, and adaptation strategies were chosen for the current investigation into copper tolerance within phylum Tardigrada. Notably, *H. crispae* was sampled at a time of year, when the population is predominantly represented by the so-called pseudo-simplex 1 stage, characterized by a doubled-layered cuticle and closed mouth and cloaca (e.g., Halberg et al., 2013c), whereas the other three species were exposed to copper in their normal active state.

In order to get a rough estimate of the expected tolerance level, we estimated the body volumes of the tardigrades to be 0.9–4.7 nl (Table 1). Based on a general relation between body size and copper-tolerance (Grosell et al., 2007), we subsequently estimated an expected EC50 for copper in the range of 0.5–2 μg l^−1^ (assuming a body density close to water, i.e., 1 μg nl^−1^). Noticeably, we found that the water from Lynæs and Vellerup Vig contained copper at concentrations of 6–7 μg l^−1^, indicating that at least the marine species would have a greater tolerance level than what would be expected from size alone.

**Table 1:** **Body volume measurements for the four species of tardigrades investigated**.

**Species (number of animals)**	**Body volume (nl) (*mean* ±*s.e.m*.)**	**Class**	**Habitat**
*E. sigismundi* (*n* = 5)	0.9 ± 0.1	Heterotardigrada	Marine
*E. testudo* (*n* = 5)	2.6 ± 0.4	Heterotardigrada	Limno-terrestrial
*R. oberhaeuseri* (*n* = 5)	4.7 ± 1.0	Eutardigrada	Limno-terrestrial
*H. crispae* (*n* = 20)	4.1 ± 0.3^*^	Eutardigrada	Marine

* *From Halberg et al. (2013b)*.

We exposed groups of ~20 tardigrades for 24 h of measured copper concentrations of up to 2991 μg l^−1^ in *E. sigismundi*, 3897 μg l^−1^ in *E. testudo*, 3065 μg l^−1^ in *H. crispae* and 3643 μg l^−1^ in *R. oberhaeuseri* (Figure 1). When dissolved in locality seawater, copper saturation seemed to be reached at a measured concentration of 3022–3376 μg l^−1^. EC50-values were obtained for two of the investigated species. Specifically, 24 h after exposures, EC50-values (95% confidence interval) were estimated at 178 (168–186) μg l^−1^ for specimens of the smallest tardigrade, *E. sigismundi*, and at 310 (295–328) μg l^−1^ for specimens of the largest species, *R. oberhaeuseri* (Figures 1B,D). Thus, in these two species, representing, respectively, Heterotardigrada and Eutardigrada as well as marine and limno-terrestrial habitats, an acute exposure to high copper concentrations seemed to be lethal. It should be noted that our data likely reflect an underestimate of survival rates as inactive tardigrades may not necessarily be dead. Table 2 summarizes EC50 values calculated at all activity assessments, i.e., at 2 h, 24 h (see also Figures 1B–E) and 48 h after exposure. Notably, the limno-terrestrial heterotardigrade *E. testudo*, and the marine eutardigrade *H. crispae* were even less affected by the metal and EC50 values could not be obtained for these species (Table 2; Figures 1C,E). The activity in percentage of *E. testudo* was 20 ± 4% (*mean* ± *s.e.m*., *n* = 4) 24 h after removal from the solutions with the highest measured copper concentrations ranging from 3,540 to 3,897 μg Cu l^−1^ (Figure 1C). Notably, activity was very high for *H. crispae*, i.e., a *mean* ± *s.e.m*. activity of 77 ± 2% (*n* = 3) was recorded 24 h after removal from the solutions with the highest copper concentrations ranging from 2,979 to 3,065 μg l^−1^ (Figure 1E). A second set of experiments was performed, in order to investigate whether copper exposure would influence longer term activity in the *H. crispae* (Figure 1F). Specifically, *H. crispae* was exposed to copper for 24 h at concentrations (379–574 μg l^−1^) that had a clear effect on *E. sigismundi* and *R. oberhaeuseri* (Figures 1B,D). Whereas, initial activity of *H. crispae* was clearly affected by exposure to copper concentrations above 500 μg l^−1^, longer term activity did not seem to be additionally affected, when compared to controls and specimens exposed to 379–490 μg Cu l^−1^. This is consistent with the “make-or-break” response observed in tardigrade radiation studies, where stress either causes animals to die during the stressful conditions or survive with no further adverse effects (e.g., Beltrán-Pardo et al., 2013, 2015).

**Table 2:** **EC50 values following copper exposure in the four species of tardigrades**.

**Species**	**EC50, 2 h (μg l^−1^)**	**EC50, 24 h (μg l^−1^)**	**EC50, 48 h (μg l^−1^)**
*E. sigismundi*	237 (228–240); *r*^2^ = 0.99	178 (168–186); *r*^2^ = 0.99	185 (177–192); *r*^2^ = 1.00
*E. testudo*	N/A; *r*^2^ = 0.56	N/A; *r*^2^ = 0.65	N/A; *r*^2^ = 0.55
*R. oberhaeuseri*	365 (343–401); *r*^2^ = 0.98	310 (295–328); *r*^2^ = 0.99	260 (233–284); *r*^2^ = 0.97
*H. crispae* (P1)	N/A; *r*^2^ = 0.18	N/A; *r*^2^ = 0.59	N/A; *r*^2^ = 0.75

*EC50 values indicating the predicted concentration of copper necessary to abolish 50% of activity (95% confidence intervals in parentheses) at the given time point following copper exposure. r^2^ values were derived from dose-response regression analyses. N/A, not applicable*.

### Putative molecular mechanisms underlying copper toxicity and tolerance

The obtained *E. sigismundi* transcriptome data was subjected to a CEGMA analysis using the CEGMA VM compilation in order to verify the quality of the assembly (Parra et al., 2007). This analysis revealed that of 248 ultra-conserved eucaryotic genes, 233 were found as complete gene sequences and 7 were found as partial gene sequences. This gives a recovery percentage of 96.8%, which is comparable to other published transcriptomes, and verifies that the obtained transcriptome database is of high quality. We screened this transcriptome for putative copper tolerance related genes.

Copper enacts toxic effects on aquatic animals by interfering with osmoregulation and excretion and by inducing oxidative stress. We, therefore, analyzed whether transcripts of well-known copper-sensitive osmoregulatory enzymes are present in the *E. sigismundi* transcriptome (Table 3). Specifically, predicted α and β subunits of the Na/K ATPase as well as carbonic anhydrase are highly expressed in this heterotardigrade and they are also present in the genome of the eutardigrade, *H. dujardini* (see Koutsovoulos et al., 2016). Furthermore, we found several transcripts, of antioxidant enzymes, which appear to be highly expressed in *E. sigismundi*, i.e., Cu-Zn superoxide dismutases, a Mn superoxide dismutase, glutathione reductase and glutathione peroxidase. The results of the multiple alignments, using CLUSTALW (Thompson et al., 1994), showed high similarity between the protein sequences of the retrieved *E. sigismundi* transcripts (Table 3) and the respective sequences of *H. dujardini* (Koutsovoulos et al., 2016), *D. melanogaster* (Flybase, Attrill et al., 2016), and other invertebrates (Genbank best hit results). It should be noted that transcripts identified as glutathione S-transferase were abundant in the *E. sigismundi* transcriptome (31 transcripts>100 bps long, with more than 100 uniquely mapped fragments, and FPKM values ranging between 9.9904 and 1882.9161, FPKM cutoff value = 1.5). Curiously, we had no annotated sequences of “catalase” in our transcriptome and the reciprocal BLAST searches did not give back any results either. We, furthermore, screened the transcriptome for transporters involved in copper homeostasis and identified two transcripts of a putative CTR copper transporter along with transcripts of a Cu-ATPase and the Cu-ATPase specific chaperone, ATOX1. No significant blast results were retrieved from the *E. sigismundi* transcriptome in the search for “metallothionein” (*e* > 0.00001). Overall, the results obtained from the current *E. sigismundi* transcriptome analyses are in congruence with data obtained from the eutardigrade species *H. dujardini*, indicating similar molecular mechanisms across different evolutionary lineages. The new *E. sigismundi* sequences (Table 3) are part of the registered Genbank BioProject ID PRJNA357357 and can also be found in the supplementary material (Supplement Table 1).

**Table 3:** **Putative copper stress related tardigrade transcripts**.

	**Transcript ID**	**BLASTX results**			**BLASTP results**	**Predicted function**
	***E. sigismundi***	**GB**	**Hd**	**Swissprot-ID**	**Pfam**	
**Osmoregulatory enzymes**	**CL1316.Contig3_ Es**	Arthropoda [AIM43570.1]	**nHd.2.3.1.t06036-RA**	P13607|ATNA_DROME	PF00122.18|E1-E2_ATPase	**Na/K ATPase subunit alpha**
	(a) 4722; (b) 4983; (c) 52.5341	(I = 78%; Eval = 0.0; Qc = 62%)	(I = 71%; Eval = 0.0)	(I = 75%; Eval = 0.0)	(Eval = 8.7e-57)	
	**CL1648.Contig1_ Es**	Arthropoda [XP_008556879.1]	**nHd.2.3.1.t12553-RA**	Q24048|ATPB2_DROME	PF00287.16|Na_K-ATPase	**Na/K ATPase subunit beta-2**
	(a) 2236; (b) 8266; (c) 184.0348	(I = 39%; Eval = 4e-58; Qc = 40%)	(I = 40%; Eval = 4e-55)	(I = 37%; Eval = 2e-53)	(Eval = 2.7e-69)	
	**CL3019.Contig1_ Es**	Arthropoda [XP_013777310.1]	**nHd.2.3.1.t03974-RA**	Q9ULX7|CAH14_HUMAN	PF00194.19|Carb_anhydrase	**Carbonic anhydrase**
	(a) 1329; (b) 4611; (c) 172.7215	(I = 40%; Eval = 9e-60; Qc = 60%)	(I = 39%; Eval = 1e-41)	(I = 39%; Eval = 9e-46)	(Eval = 6.9e-75)	
	**Unigene4595_ Es**	Arthropoda [JAN40855]	**nHd.2.3.1.t13678-RA**	Q22460|BCA1_CAEEL	PF00484.17|Pro_CA	**Beta carbonic anhydrase**
	(a) 1137; (b) 1554; (c) 68.0404	(I = 54%; Eval = 6e-85; Qc = 64%)	(I = 32%; Eval = 4e-31)	(I = 43%; Eval = 4e-51)	(Eval = 4e-51)	
**Antioxidant enzymes**	**Unigene7967_ Es**	Arthropoda [XP_013794733.1]	**nHd.2.3.1.t08435-RA**	Q55GQ5|SODC1_DICDI	PF00080.18|Sod_Cu	**Cu-Zn superoxide dismutase**
	(a) 1089; (b) 1546; (c) 70.6737	(I = 54%; Eval = 5e-49; Qc = 41%)	(I = 49%; Eval = 5e-39)	(I = 52; Eval = 3e-42)	(Eval = 2.0e-44)	
	**Unigene7176_ Es**	Mollusca [AET43974.1]	**nHd.2.3.1.t08389-RA**	Q8HXP5|SODM_HYLLA	PF02777.16|Sod_Fe_C	**Mn Superoxide dismutase**
	(a) 825; (b) 2116; (c) 127.6845	(I = 58%; Eval = 9e-93; Qc = 82%)	(I = 59%; Eval = 8e-72)	(I = 60%; Eval = 9e-71)	(Eval = 8.4e-35)	
	**Unigene9127_ Es**	Mollusca [XP_005099721.2]	**nHd.2.3.1.t10722-RA**	P47791|GSHR_MOUSE	PF07992.12|Pyr_redox_2	**Glutathione reductase**
	(a) 1619; (b) 3020; (c) 92.8617	(I = 62%; Eval = 0.0; Qc = 83%)	(I = 56%; Eval = 2e-50)	(I = 59%; Eval = 2e-156)	(Eval = 2e-156)	
	**CL47.Contig3_ Es**	Rotifera [AIL94180.1]	**nHd.2.3.1.t11424-RA**	Q00277|GPX1_SCHMA	PF00255.17|GSHPx	**Gluthathione peroxidase**
	(a) 1291; (b) 4002; (c) 154.3218	(I = 58%; Eval = 2e-68; Qc = 42%)	(I = 51%; Eval = 5e-52)	(I = 73%; Eval 2e-18)	(Eval = 4.0e-44)	
**Copper transport**	**Unigene8200_ Es**	Annelida [ELT91953.1]	**nHd.2.3.1.t02534-RA**	Q8K211|COPT1_MOUSE	PF04145.13|Ctr	**Ctr copper transporter**
	(a) 975; (b) 1440; (c) 73.5249	(I = 38%; Eval = 3e-32; Qc = 59%)	(I = 28%; Eval = 5e-10)	(I = 32%; Eval = 1e-20)	(Eval = 2.2e-28)	
	**Unigene10756_ Es**	Mollusca [AEJ08756.1]	**nHd.2.3.1.t04635-RA**	Q55BB8|COG8_DICDI	PF00403.24|HMA	**Copper chaperone Atox1**
	(a) 1607; (b) 597; (c) 18.4942	(I = 51%; Eval = 1e-15; Qc = 12%)	(I = 42%; Eval = 5e-7)	(I 23%; Eval = 2e-8)	(Eval = 1.2e-11)	
	**Unigene12304_ Es**	Arthropoda [XP_015601105.1]	**nHd.2.3.1.t07554-RA**	Q04656|ATP7A_HUMAN	PF00403.24|HMA	**Copper-transporting ATPase**
	(a) 3397; (b) 3599; (c) 52.7427	(I = 51%; Eval = 0.0; Qc = 87%)	(I = 49%; Eval = 1e-159)	(I = 49%; Eval = 0.0)	(Eval = 2.2e-14)	

*E. sigismundi transcripts with information on (a) sequence length (bp), (b) number of raw fragments, (c) FPKM value E. sigismundi transcript sequences are provided in Supplement Table 1. BLASTX best hit results of each E. sigismundi transcript against NCBI (GB), H. dujardini genome (Hd) (http://badger.bio.ed.ac.uk/H_dujardini/), and Swissprot database with the percentage of identity (I), e-value (Eval) and, when provided, query coverage (Qc) within parenthesis. BLASTP best hit results from the Pfam database. Tardigrade transcripts and proposed functions are given in bold*.

## Discussion

Our copper tolerance assay revealed that tardigrades have a much higher tolerance toward the toxicant than what would be expected from their body size alone (Table 1; Grosell et al., 2007). For comparison, the less tolerant tardigrade species, *E. sigismundi* and *R. oberhauseri*, from which EC50-values could be obtained (Figures 1B,D; Table 2), had considerable higher tolerance than the much larger species of the freshwater mussel family, Unionidae, with reported EC50-values (48 h exposure) in the range of 14–57 μg l^−1^ (Wang et al., 2007). The tardigrade EC50-values were, however, within the range of the well-established bioindicator species, *D. magna*, which is somewhat larger than the tardigrades and has a reported EC50 (48 h exposure, pH 7.08) of ~424 μg l^−1^ (De Schamphelaere et al., 2002). Notably, two of the tardigrade species, *E. testudo* and *H. crispae*, had even higher tolerance (Figures 1C,E) with no obvious EC50-values. All tardigrade species seem to have significantly higher tolerance toward copper than that reported from *A. franciscana* with a 48 h EC50 of 68.3 μg l^−1^ based on hatching success (Brix et al., 2006).

Our results indicate that copper tolerance neither reflects the different evolutionary lineages within the tardigrades nor their habitat. It is, however, highly possible that the double cuticle of the *H. crispae* P1 stage protects against copper toxicity, e.g., by sequestering the toxicant, which would explain the extremely high tolerance found for this species. The latter would suggest that cysts, which are widespread within the phylum, have an increased tolerance toward toxicants. Tardigrades are renowned for their ability to depress metabolism and, besides cyst formation, they may enter the ametabolic state of cryptobiosis in response to environmental extremes (Guidetti et al., 2011; Møbjerg et al., 2011; Welnicz et al., 2011). Importantly, increased activity rates were recorded after exposure to the highest copper concentrations in both *H. crispae* and *E. testudo* (Figures 1C,E), which could indicate that high toxicant concentrations can induce the highly resilient and quiescence state of chemobiosis, i.e., cryptobiosis induced by toxins and toxicants (Møbjerg et al., 2011). Further, investigations are, however, necessary in order to firmly establish whether acclimation to copper and other toxicants can induce such a state.

Notably, transcripts of well-known copper-sensitive osmoregulatory enzymes were present in the *E. sigismundi* transcriptome. Specifically, α and β subunits of the Na/K ATPase as well as carbonic anhydrase seem highly expressed in this heterotardigrade and sequences of these enzymes are also present in the eutardigrade *H. dujardini* (Table 3). Thus, lethal effects of high copper concentrations on active tardigrades could be caused by interference with these copper sensitive key osmoregulatory enzymes. As has been proposed for the extraordinary radiation tolerance seen among tardigrades (Jönsson, 2003; Jönsson et al., 2005; Horikawa et al., 2006, 2013; Altiero et al., 2011; Beltrán-Pardo et al., 2015; Hashimoto et al., 2016), high toxicant tolerance could relate to molecular mechanisms (e.g., a well-developed antioxidant defense system) that evolved in relation to cryptobiosis. Moreover, if tardigrades indeed have the ability to suppress metabolism and enter cryptobiosis in response to high toxicant concentrations (i.e., chemobiosis), this would abolish any deleterious effects of the toxicant on active osmoregulation and at the same time decrease the production of the free radicals deriving from energy turnover, thereby limiting cellular and genomic damage.

High concentrations of copper may, through binding to various proteins, lead to increased production of highly reactive oxygen species. Antioxidant proteins are therefore essential in minimizing concurrent oxidative stress damage (Bertinato and L'Abbé, 2004; Egli et al., 2006). Notably, previous studies have shown that tardigrades have the ability to upregulate antioxidant defense systems in connection with cryptobiosis (Mali et al., 2010; Rizzo et al., 2010) and furthermore that superoxide dismutases, among other stress-related gene families, are expanded in tardigrades (Hashimoto et al., 2016). We found several transcripts of superoxide dismutases, which appear to be highly expressed in *E. sigismundi* and show a high similarity to the protein sequence of the homolog genes in *H. dujardini* and *D. melanogaster* (Table 3). In addition, the *E. sigismundi* transcriptome contains transcripts of glutathione reductase, glutathione peroxidase and glutathione S transferase. Thus, the genetic basis of pathways able to handle free radicals is clearly present and highly expressed in *E. sigismundi*. Moreover, copper is likely excreted or could, analogous to e.g., *Drosophila* cuprophilic cells (Filshie et al., 1971), be sequestered in specific cells (e.g., tardigrade body cavity cells). Along this line, *E. sigismundi* expresses a putative CTR copper transporter, likely involved in cellular copper uptake. Importantly, transcripts were also present of a cytosolic ATOX1 copper carrier, and a copper efflux Cu-ATPase. Thus, as has been described from various vertebrates (e.g., Gupta and Lutsenko, 2009; Minghetti et al., 2010), tardigrades seem to have the cellular machinery enabling copper sequestering and excretion. During a copper load, copper could thus enter the cell through the CTR transporter, and upon release be accepted by the ATOX1, which would then transfer copper to the Cu-ATPase for delivery into e.g., a secretory vesicle. Our search for the metal sequestering metallothioneins did not reveal any transcripts in the *E. sigismundi* transcriptome, which could be explained by the high heterogeneity of these sequences.

In summary, our results show that tardigrades have high tolerance toward copper, likely linked to a well-developed battery of transporters involved in copper homeostasis, a high expression of antioxidant enzymes and the ability to enter dormant states. The current study presents a novel tolerance assay, which can be used in future investigations on tardigrade toxicant tolerance. Furthermore, it presents novel sequence data from the tardigrade *E. sigismundi* with a phylogenetic position, which could hold key evolutionary evidence to how heterotardigrades colonized terrestrial environments (Møbjerg et al., 2011, 2016; Faurby et al., 2012; Hygum et al., 2016).

## Author contributions

AJ, MG, and NM conceived and designed the study. AJ, DF, TH, and NM collected and sorted tardigrades. MG measured copper concentrations. DF and TH carried out the copper tolerance assays. MS and NM prepared the RNA for RNA-seq. DF, MK, MS, and TH retrieved data from the transcriptome. MK performed the comparative transcriptome analysis and MS the CEGMA analysis. TH and NM wrote the manuscript with inputs from the other authors.

## Funding

The study was funded by The Danish Council for Independent Research (grant-ID: DFF–4090-00145 to NM) and the Carlsberg Foundation. MG is a Maytag Professor of Ichthyology.

### Conflict of interest statement

The authors declare that the research was conducted in the absence of any commercial or financial relationships that could be construed as a potential conflict of interest. The reviewer RM and handling Editor declared their shared affiliation, and the handling Editor states that the process nevertheless met the standards of a fair and objective review.
